# Tradeoffs between targeted and expanded testing strategies for influenza A(H5N1)

**DOI:** 10.1128/asmcr.00053-26

**Published:** 2026-06-24

**Authors:** Daniel A. Green

**Affiliations:** 1 Department of Pathology & Cell Biology, Columbia University Vagelos College of Physicians and Surgeons 12294, New York, New York, USA; Pattern Bioscience, Austin, Texas, USA

**Keywords:** avian influenza, influenza, H5N1, HPAI, laboratory preparedness

## Abstract

In a recent article in *ASM Case Reports*, G. P. Higerd-Rusli, A. Karan, S. A. Hoffman, I. E. A. Morante, et al. (ASM Case Rep 2:e00165-25, 2025, https://doi.org/10.1128/asmcr.00165-25) describe one confirmed and one probable case of influenza A(H5N1) detected through universal subtyping of influenza A-positive specimens, including cases without known animal exposure. These findings highlight the limitations of exposure-based testing strategies and raise important questions about early detection of emerging pathogens. Drawing on lessons from prior pandemics, this commentary examines the tradeoffs between targeted and expanded surveillance and emphasizes the need for adaptive, flexible approaches to laboratory preparedness.

## COMMENTARY

The epidemiology of highly pathogenic avian influenza A(H5N1) has shifted substantially in the past 2 years (1). Once largely confined to avian hosts, outbreaks have now been documented across multiple mammalian species, most notably among dairy cattle in the United States (2). Dozens of human infections have occurred through contact with infected animals (3), with the prospect of sustained human-to-human transmission an ever-present concern. To date, however, human testing has been limited to surveillance of individuals with known exposures to infected animals.

A recent study by Higerd-Rusli et al. in *ASM Case Reports* highlights concerns with this testing approach (4). The authors describe one confirmed and one probable case of influenza A(H5N1) identified through universal H5 subtyping of all influenza A-positive respiratory specimens at a large academic healthcare system in California. Sequencing of the confirmed case showed that it was phylogenetically related to clade 2.3.4.4b viruses associated with dairy cattle. The probable case showed reproducible detection of H5 at high cycle threshold values but could not be confirmed by the state public health laboratory or the Centers for Disease Control and Prevention (CDC). In both cases, the patients had no known exposures to infected animals, underscoring that neither case would have been captured by exposure-based testing strategies.

Current testing and surveillance strategies are targeted to individuals with the highest risk of disease. At present, the CDC recommends symptomatic monitoring for persons exposed to confirmed H5N1 infections in humans or animals (5). Those who develop compatible signs and symptoms should then undergo testing. While this strategy is practical and resource-conscious, it assumes that exposures are promptly identified and that transmission pathways remain stable and predictable.

The two cases presented here underscore the limitations of these assumptions and demonstrate the existence of edge cases that fall outside traditional testing paradigms. It should be emphasized that the detection of H5 in these two patients in itself does not establish community transmission, as these cases could simply reflect indirect/unknown exposures or environmental contamination. The authors even speculate whether consumption of dairy milk could have contaminated respiratory specimens with H5 viral RNA. Furthermore, the very low positivity rate reported by the authors (0.04%) is reassuring in that community transmission of H5N1 does not appear widespread. However, these two cases should nevertheless jolt readers into recognizing that the full extent of these and similar cases remains unknowable within current surveillance frameworks.

The debate about targeted vs. expanded surveillance for emerging high-consequence pathogens is not new. During the 2009 influenza A H1N1 pandemic, testing initially focused on severe cases, which led to under-recognition of milder infections (6, 7). Only after testing was broadened was the full scope of transmission more clearly appreciated. Similarly, early SARS-CoV-2 testing was limited and restricted to patients with known travel or exposures, delaying recognition of asymptomatic spread and community transmission (8, 9). Subsequent analyses demonstrated that widespread transmission had already occurred but went undetected due to restrictive case definitions for testing, which reduced the effectiveness of contact tracing and containment strategies (9–11).

What lessons can be learned then from previous spillover events, and how can these experiences inform future testing strategies for pathogens with pandemic potential like influenza A(H5N1)? Any expansion of testing must consider the diagnostic tradeoffs. The probable case reported by the authors illustrates the challenges of test interpretation in a low-prevalence setting. Detection of viral RNA near the limit of detection raises concerns about assay specificity, reproducibility, and the potential for false positives. Similar challenges were encountered during the SARS-CoV-2 pandemic, where high cycle threshold detections required careful interpretation in low-prevalence settings (12). Given the high sensitivity of molecular assays, expanding surveillance too early or in the wrong populations can potentially generate misleading results and complicate infection control efforts.

Broader testing also requires substantial resources and investment, which may be difficult to justify in the setting of workforce and funding constraints. There are also important questions of where and how expanded surveillance should be performed. While most clinical laboratories can detect influenza A, there are no widely available FDA-cleared assays for H5 subtyping. The authors of this report developed their own dual-target PCR assay (13), while the CDC also manufactures and licenses a four-target PCR assay that it provides to state public health laboratories (14). However, laboratory-developed tests (LDTs) like these, especially those with a higher number of targets, are limited in their scalability for high-volume testing (14). Their implementation also requires technical expertise, extensive validation, and ongoing quality assurance. Regulatory requirements may further limit adoption in certain states.

It is also notable that these cases were identified at a large academic medical center rather than through public health surveillance systems. The authors’ proactive approach, while commendable, may not fall under the purview of most clinical laboratories. Coupled with the limitations of scaling LDTs, broader testing in clinical laboratories is unlikely to meaningfully expand until high-throughput tests become available through FDA authorization pathways. Until then, subtyping is more likely to be scaled up within state public health laboratories. However, reliance on a single CDC-developed assay also brings its own vulnerabilities, as illustrated by the assay-related issues that led to delayed public health testing for SARS-CoV-2 (15).

Collectively, these considerations highlight the fundamental challenges and tradeoffs inherent to laboratory preparedness for emerging high-consequence pathogens (Table 1). Expanding testing too early risks overinvestment of resources with limited yield and a higher likelihood of misleading results (16, 17). Waiting too long, on the contrary, risks allowing transmission to become more firmly established before it can be recognized, which may render early containment strategies substantially less effective (8–11, 18). These competing priorities create an obvious dilemma: early cases of community transmission may only be detected once testing is broadened, but the decision to broaden testing depends on detecting these exact types of cases.

**TABLE 1:** Tradeoffs between targeted and expanded surveillance for influenza A(H5N1)

	Targeted (exposure-based) surveillance	Expanded (universal or broad) surveillance
Testing population	Individuals with known animal exposure and compatible symptoms	All influenza A-positive specimens or broader populations, regardless of exposure
Operational setting	Primarily public health-driven	Requires closer coordination between clinical and public health laboratories
Resource utilization	Efficient; conserves laboratory and public health resources	Resource-intensive; requires staffing, reagents, and infrastructure
Scalability	Scalable within existing workflows	More limited by assay availability, throughput, and validation requirements
Sensitivity for early/atypical cases	Lower; may miss cases without recognized exposure	Higher; enables detection of unexpected or cryptic infections
Detection of community transmission	Delayed; may miss early spread outside known exposure networks	Earlier detection possible
Specificity/interpretability	Higher pretest probability; results easier to interpret	Lower pretest probability; increased risk of ambiguous or false positive results
Dependence on epidemiologic assumptions	High; relies on accurate identification of exposures and transmission pathways	Lower; less dependent on recognizing exposures in advance
Timing/trigger for implementation	Implemented early based on known risk pathways	Typically implemented later once broader transmission justifies expansion

In this context, geographically targeted or hybrid surveillance strategies, as suggested by the authors, may represent a practical middle ground (4). Focusing enhanced subtyping in regions with known animal outbreaks, high-risk agricultural interfaces, or early signals of atypical influenza activity could improve detection while preserving laboratory capacity and diagnostic specificity. Most countries have relied on exposure-based testing strategies for high-consequence infectious diseases, including Ebola, MERS-CoV, and early SARS-CoV-2 (11, 19). In contrast, expanded testing approaches in some countries during the COVID-19 pandemic demonstrated broader detection but proved resource-intensive and difficult to sustain (20, 21), supporting targeted or hybrid expansion as a more practical path forward.

Ultimately, the question is not whether all laboratories should adopt universal H5 subtyping, but how surveillance systems can remain sensitive to detect early outbreaks without overextending limited resources. Experience from prior pandemics suggests that testing often expands reactively, after transmission is already established. The findings presented here argue for a more proactive and adaptive approach, one that anticipates the limitations of exposure-based strategies and incorporates more flexibility in laboratory preparedness.

## References

[1] Krammer F, Hermann E, Rasmussen AL. 2025. Highly pathogenic avian influenza H5N1: history, current situation, and outlook. J Virol 99:e0220924. doi:10.1128/jvi.02209-24 40145745 PMC11998540

[2] Nguyen T-Q, Hutter CR, Markin A, Thomas M, Lantz K, Killian ML, Janzen GM, Vijendran S, Wagle S, Inderski B, et al.. 2025. Emergence and interstate spread of highly pathogenic avian influenza A(H5N1) in dairy cattle in the United States. Science 388:eadq0900. doi:10.1126/science.adq0900 40273240

[3] CDC. 2025. H5 bird flu: current situation. avian influenza bird flu. Available from: https://www.cdc.gov/bird-flu/situation-summary/index.html

[4] Higerd-Rusli GP, Karan A, Hoffman SA, Morante IEA, Huang C, Sahoo MK, Hernandez MM, Pinsky BA. 2025. One confirmed and one potential human case of influenza A(H5N1) detected through an expanded subtyping protocol. ASM Case Rep 2:e00165-25. doi:10.1128/asmcr.00165-25 PMC12772291 41503532

[5] CDC. 2025. Interim guidance on specimen collection and testing for patients with suspected infection with Novel Influenza a viruses associated with severe disease or with the potential to cause severe disease in humans. Available from: https://www.cdc.gov/bird-flu/php/severe-potential/index.html

[6] Jernigan DB, Lindstrom SL, Johnson JR, Miller JD, Hoelscher M, Humes R, Shively R, Brammer L, Burke SA, Villanueva JM, Balish A, Uyeki T, Mustaquim D, Bishop A, Handsfield JH, Astles R, Xu X, Klimov AI, Cox NJ, Shaw MW. 2011. Detecting 2009 pandemic influenza A (H1N1) virus infection: availability of diagnostic testing led to rapid pandemic response. Clin Infect Dis 52 Suppl 1:S36–43. doi:10.1093/cid/ciq020 21342897

[7] Reed C, Angulo FJ, Swerdlow DL, Lipsitch M, Meltzer MI, Jernigan D, Finelli L. 2099. Estimates of the prevalence of pandemic (H1N1) 2009, United States, April-July 2009. Emerg Infect Dis 15:2004–2007. doi:10.3201/eid1512.091413 PMC3375879 19961687

[8] Ghinai I, Woods S, Ritger KA, McPherson TD, Black SR, Sparrow L, Fricchione MJ, Kerins JL, Pacilli M, Ruestow PS, Arwady MA, Beavers SF, Payne DC, Kirking HL, Layden JE. 2020. Community transmission of SARS-CoV-2 at two family gatherings - Chicago, Illinois, February-March 2020. MMWR Morb Mortal Wkly Rep 69:446–450. doi:10.15585/mmwr.mm6915e1 32298246 PMC7755060

[9] Worobey M, Pekar J, Larsen BB, Nelson MI, Hill V, Joy JB, Rambaut A, Suchard MA, Wertheim JO, Lemey P. 2020. The emergence of SARS-CoV-2 in Europe and North America. Science 370:564–570. doi:10.1126/science.abc8169 32912998 PMC7810038

[10] Chappell JG, Tsoleridis T, Clark G, Berry L, Holmes N, Moore C, Carlile M, Sang F, Debebe BJ, Wright V, Irving WL, Thomson BJ, Boswell TCJ, Willingham I, Joseph A, Smith W, Khakh M, Fleming VM, Lister MM, Howson-Wells HC, Holmes EC, Loose MW, Ball JK, McClure CP. 2021. Retrospective screening of routine respiratory samples revealed undetected community transmission and missed intervention opportunities for SARS-CoV-2 in the United Kingdom. J Gen Virol 102:001595. doi:10.1099/jgv.0.001595 34130773 PMC8459093

[11] Hellewell J, Abbott S, Gimma A, Bosse NI, Jarvis CI, Russell TW, Munday JD, Kucharski AJ, Edmunds WJ, Centre for the Mathematical Modelling of Infectious Diseases COVID-19 Working Group, Funk S, Eggo RM. 2020. Feasibility of controlling COVID-19 outbreaks by isolation of cases and contacts. Lancet Glob Health 8:e488–e496. doi:10.1016/S2214-109X(20)30074-7 32119825 PMC7097845

[12] Rhoads D, Peaper DR, She RC, Nolte FS, Wojewoda CM, Anderson NW, Pritt BS. 2021. College of American Pathologists (CAP) microbiology committee perspective: caution must be used in interpreting the Cycle Threshold (Ct) value. Clin Infect Dis 72:e685–e686. doi:10.1093/cid/ciaa1199 32785682

[13] Sahoo MK, Morante IEA, Huang C, Solis D, Yamamoto F, Ohiri UC, Romero D, Pinsky BA. 2024. Multiplex dual-target reverse transcription PCR for subtyping avian influenza A(H5) virus. Emerg Infect Dis 30:1710–1713. doi:10.3201/eid3008.240785 38986151 PMC11286043

[14] Pinsky BA, Bradley BT. 2024. Opportunities and challenges for the U.S. laboratory response to highly pathogenic avian influenza A(H5N1). J Clin Virol 174:105723. doi:10.1016/j.jcv.2024.105723 39213758

[15] Grimm CA. 2023. CDC’s internal control weaknesses led to its initial COVID-19 test kit failure, but CDC ultimately created a working test kit. Available from: https://oig.hhs.gov/oas/reports/region4/42002027.asp

[16] Surkova E, Nikolayevskyy V, Drobniewski F. 2020. False-positive COVID-19 results: hidden problems and costs. Lancet Respir Med 8:1167–1168. doi:10.1016/S2213-2600(20)30453-7 33007240 PMC7524437

[17] Altman DG, Bland JM. 1994. Diagnostic tests. 1: sensitivity and specificity. BMJ 308:1552. doi:10.1136/bmj.308.6943.1552 8019315 PMC2540489

[18] Mina MJ, Parker R, Larremore DB. 2020. Rethinking Covid-19 test sensitivity - a strategy for containment. N Engl J Med 383:e120. doi:10.1056/NEJMp2025631 32997903

[19] Guy D, Kodjamanova P, Woldmann L, Sahota J, Bannister-Tyrrell M, Elouard Y, Degail MA. 2025. Contact tracing strategies for infectious diseases: a systematic literature review. PLoS Glob Public Health 5:e0004579. doi:10.1371/journal.pgph.0004579 40343962 PMC12063836

[20] Peto J, Alwan NA, Godfrey KM, Burgess RA, Hunter DJ, Riboli E, Romer P, 27 signatories. 2020. Universal weekly testing as the UK COVID-19 lockdown exit strategy. Lancet 395:1420–1421. doi:10.1016/S0140-6736(20)30936-3 32325027 PMC7172826

[21] Holt E. 2020. Slovakia to test all adults for SARS-CoV-2. The Lancet 396:1386–1387. doi:10.1016/S0140-6736(20)32261-3 PMC7598435 33129382

